# Editorial: Genetic underpinnings of Alzheimer’s and Parkinson’s: insights and innovations

**DOI:** 10.3389/fgene.2026.1846361

**Published:** 2026-04-21

**Authors:** Roberta Marongiu, Dennis Cordato, Allison B. Reiss

**Affiliations:** 1 Department of Neurological Surgery, Weill Cornell Medicine, Cornell University, New York, NY, United States; 2 Feil Family Brain and Mind Institute, Weill Cornell Medicine, Cornell University, New York, NY, United States; 3 Department of Genetic Medicine, Weill Cornell Medicine, Cornell University, New York, NY, United States; 4 Department of Neurology, Liverpool Hospital, Sydney, NSW, Australia; 5 South Western Sydney Clinical School, University of NSW, Sydney, NSW, Australia; 6 Departments of Medicine and Foundations of Medicine, NYU Grossman Long Island School of Medicine, Mineola, NY, United States

**Keywords:** Alzheimer’s disease, biomarkers, GBA, LRRK2, Parkinson's disease, polygenic scores (PGS)

## Introduction

Alzheimer’s disease (AD) and Parkinson’s disease (PD) are multifactorial neurodegenerative disorders that are among the leading causes of disability and death around the globe (Alzheimer’s Association, 2023; Bloem et al., 2021; GBD, 2016 Parkinson’s Disease Collaborators, 2018). Both are incurable, progressive, have a major impact on quality of life and are increasing in prevalence (Alzheimer’s Association, 2023; GBD, 2015 Neurological Disorders Collaborator Group, 2017). Despite their distinct main clinical presentations these diseases share some common underlying biological mechanisms related to oxidative stress, neuroinflammation and mitochondrial dysfunction (Zhang et al., 2024; Poewe et al., 2017).

AD manifests primarily as memory impairment and cognitive decline (Zhang et al., 2024; Frisoni et al., 2025). While PD may bring affective disturbances and cognitive impairment, its most prominent features are motor, including resting tremor, bradykinesia, and muscular rigidity (Alzheimer’s Association, 2023). The neuropathology of AD is typically seen under the microscope as intracellular neurofibrillary tangles consisting of phosphorylated tau protein and excessive accumulation of amyloid-β in extracellular plaques (Zhang et al., 2024). Pathologically, the hallmarks of PD are selective loss of dopaminergic neurons in the substantia nigra region of the brain and the misfolding of alpha-synuclein with eventual Lewy body formation (Poewe et al., 2017). Both can be influenced by genetics, but also occur without any identifiable hereditary component. Disease-modifying treatment options are currently limited for both.

Genetic factors have long been recognized as contributors to both AD and PD, but their role extends beyond conferring susceptibility. Increasing evidence suggests that genetic architecture shapes not only disease risk, but also the timing of onset, progression dynamics, and the emergence of specific clinical features, including cognitive and neuropsychiatric manifestations. In this context, genetics provides a critical framework through which to reinterpret neurodegeneration—not as a static endpoint, but as a dynamic and individualized process.

## The special Research Topic

This Special Research Topic brings together a set of studies that collectively advance this perspective, highlighting how genetic variation intersects with molecular pathways, biomarkers, and clinical phenotypes to refine our understanding of AD and PD. A unifying theme emerging from this Research Topic is that genetic variation acts as a modifier of disease trajectory, influencing both biological mechanisms and clinical expression across the disease continuum.

Ikanga et al. provide insight into the clinical utility of plasma AD biomarkers, including amyloid β (Aβ42/40), phosphorylated tau, neurofilament light (Nfl), glial fibrillary acid protein (GFAP) and tumor necrosis factor α in an underrepresented population of individuals from the Democratic Republic of Congo. This is not only the first study to provide reference values for plasma AD biomarkers in sub-Saharan Africa, but also establishes that plasma AD biomarkers are a potentially cost-effective and easily accessible screening tool in resource-limited populations in which genetic testing and imaging biomarker studies may be unavailable or unaffordable. Further research is needed to explore the predictive value of plasma biomarkers in various ethnically and culturally diverse populations—an essential step toward equitable implementation of precision medicine.

Polygenic scores (PGSs) are becoming increasingly recognized as crucial tools for assessing an individual’s genetic risk in AD. Mwesigwa et al., address a growing challenge in AD research—the fragmentation and limited usability of polygenic score datasets. By developing a systematic framework for the curation and annotation of PGSs, the authors polygenic risk as an integrative tool capable of linking genetic variation to biological pathways and disease mechanisms. As datasets continue to expand, such harmonization efforts will be essential to translate polygenic risk into clinically actionable insights.

Lin et al. explore the role of nicotine and nicotinic acetylcholine receptors (nAChRs) in the pathophysiology of PD. Their review revisits the role of nAChRs in PD through a genetic and systems lens. Their synthesis positions nAChRs at the intersection of mitochondrial function, oxidative stress, synaptic signaling, and neuroprotection in dopaminergic neurons^,^ suggesting that receptor-level dynamics may represent a convergence point between environmental exposures and genetic susceptibility. This perspective highlights the importance of considering gene–environment interactions within a systems framework. The role of alterations in the function and structure of nAChRs in the progression of PD, including the effects of age and the potential neuroprotective mechanisms of action of nicotine on different nAChR subunits in various PD animal models was also discussed. Further research on elucidating the role of nAChRs in genetic PD subtypes such as glucocerebrosidase and SNCA mutations is warranted.

Beyond susceptibility, other contributions in this Research Topic emphasize the role of genetic variation in shaping disease progression and clinical phenotype. Two studies further extend this framework by linking genetic variation to specific dimensions of PD progression, with particular emphasis on cognitive and neuropsychiatric outcomes.

A comprehensive meta-analysis by Li et al. demonstrates that glucocerebrosidase 1 (GBA1) gene variants substantially increase the risk of PD dementia (PDD), reinforcing lysosomal dysfunction as a key axis connecting genetic risk to neurodegenerative spread and cognitive decline. Notably, both pathogenic mutations and polymorphic variants contributed to this elevated risk, with certain mutations such as L444P conferring particularly strong effects. Although the concept of a link between GBA1 mutations and PD risk is not novel, these findings reinforce the central role of lysosomal dysfunction in PD pathogenesis and highlight GBA1 status as a key determinant not only of disease susceptibility but also of clinical trajectory, including cognitive decline. Importantly, the graded effects observed across variants suggest that genetic stratification may inform not only risk prediction but also prognosis and therapeutic targeting.

Complementing this work, Ng et al. provide compelling evidence that LRRK2 R1441G mutation exert early and domain-specific effects on affective behavior, even in the absence of overt motor or cognitive deficits. By demonstrating that the heterozygous LRRK2 R1441G knock-in mouse model presents selective neuropsychiatric phenotypes (including increased behavioral despair and anhedonia), this study supports a model in which genetic perturbations manifest along distinct functional axes, potentially long before classical clinical thresholds are reached. These findings reinforce the importance of capturing prodromal and non-motor features as integral components of genetically driven disease trajectories. The inclusion of heterozygous models—more representative of the human condition—adds significant translational relevance and underscores the importance of modeling subtle, early-stage phenotypes.

Together, these studies emphasize that genetic risk factors in PD extend beyond disease initiation to shape phenotypic heterogeneity, particularly in non-motor and cognitive domains. They also highlight the importance of integrating genetic stratification with longitudinal phenotyping to better capture the full spectrum of disease progression.

## Conclusion

AD and PD both have prolonged prodromal periods during which interventions may have the optimal chance for success. It is hoped that the original articles and reviews in this Research Topic will give the reader fresh perspectives on the interwoven and complex underlying genetic factors that lead down the path to AD and PD. Advancement toward disease-modifying therapy in one of these disorders may well catalyze parallel breakthrough in the other, particularly as shared genetic and molecular pathways continue to emerge as convergent drivers of neurodegeneration.
